# Pyroptotic Patterns in Blood Leukocytes Predict Disease Severity and Outcome in COVID-19 Patients

**DOI:** 10.3389/fimmu.2022.888661

**Published:** 2022-07-19

**Authors:** Yingkui Tang, Peidong Zhang, Qiuyu Liu, Luyang Cao, Jingsong Xu

**Affiliations:** ^1^ State Key Laboratory of Biotherapy and Cancer Center, West China Hospital, Sichuan University, Chengdu, China; ^2^ Department of Critical Care Medicine, Yongchuan Hospital, Chongqing Medical University, Chongqing, China; ^3^ Guangzhou Regenerative Medicine and Health Guangdong Laboratory (GRMH-GDL), Guangzhou, China

**Keywords:** COVID-19, pyroptosis, leukocytes, prognosis model, transcription factors

## Abstract

The global coronavirus disease 2019 (COVID-19) pandemic has lasted for over 2 years now and has already caused millions of deaths. In COVID-19, leukocyte pyroptosis has been previously associated with both beneficial and detrimental effects, so its role in the development of this disease remains controversial. Using transcriptomic data (GSE157103) of blood leukocytes from 126 acute respiratory distress syndrome patients (ARDS) with or without COVID-19, we found that COVID-19 patients present with enhanced leukocyte pyroptosis. Based on unsupervised clustering, we divided 100 COVID-19 patients into two clusters (PYRcluster1 and PYRcluster2) according to the expression of 35 pyroptosis-related genes. The results revealed distinct pyroptotic patterns associated with different leukocytes in these PYRclusters. PYRcluster1 patients were in a hyperinflammatory state and had a worse prognosis than PYRcluster2 patients. The hyperinflammation of PYRcluster1 was validated by the results of gene set enrichment analysis (GSEA) of proteomic data (MSV000085703). These differences in pyroptosis between the two PYRclusters were confirmed by the PYRscore. To improve the clinical treatment of COVID-19 patients, we used least absolute shrinkage and selection operator (LASSO) regression to construct a prognostic model based on differentially expressed genes between PYRclusters (PYRsafescore), which can be applied as an effective prognosis tool. Lastly, we explored the upstream transcription factors of different pyroptotic patterns, thereby identifying 112 compounds with potential therapeutic value in public databases.

## Introduction

The coronavirus disease 2019 (COVID-19) pandemic caused by the severe acute respiratory syndrome coronavirus 2 (SARS-CoV-2) has killed 6 million people until May 2022 (1). COVID-19 patients present with different clinical symptoms ranging from mild cold-like symptoms to a high fever, pneumonia, and possibly acute respiratory distress syndrome (ARDS). In the development of severe COVID-19 disease, uncontrolled systemic hyperinflammation caused by a dysregulated immune response leads to the release of pro-inflammatory cytokines and chemokines, a condition that is known as cytokine storm (2). COVID-19 patients show elevated blood levels of many cytokines, including IL-1β, IL-2, IL-6, IL-7, IL-8, IL-10, IL-18, G-CSF, IP-10, MCP-1, MIP-1A, and TNF (3–6). This cytokine storm is closely related to lung damage, multiple organ failure, and a poor prognosis, according to recent research (4, 7–11). Concurrently, several studies have also shown that cytokine blockade can improve the survival rate of patients with COVID-19 (12–15).

A possible mechanism linking cytokine storm to organ damage is inflammatory cell death, namely pyroptosis and necroptosis. Pyroptosis has been intensely studied recently. Some patients with severe COVID-19 may develop a systemic cytokine storm because SARS-CoV-2 promotes cytokine storms by inducing pyroptosis in pro-inflammatory blood-born immune cells (16–19). However, only a few studies about necroptosis and cytokine storm in COVID-19 have been published thus far.

Pyroptosis is a mechanism of programmed cell death characterized by the inflow of sodium ions and water mediated by gasdermin proteins, resulting in cell membrane rupture, excessive cell swelling, and spontaneous release of cytosolic contents into the extracellular space (20). Gasdermin proteins, which consist of an N-terminus with membrane pore-forming activity and an inhibitory C-terminus, are the key regulators of pyroptosis. Upon inflammasome activation, caspase proteins, including caspase-1 and other non-canonical inflammasome caspases (e.g., caspase-4, caspase-5, and caspase-11), cleave gasdermin into two parts (21), thereby unleashing the pore-forming activity of the N-terminus. This N-terminus fragment of gasdermin binds to the cell membrane, forming pores and leading to pyroptosis (22). Pyroptosis triggers the rapid release of a slew of alarmins including, cytokines (IL-1β, IL-18), chemokines, and damage-associated molecular patterns (DAMPs), prompting an immediate response from surrounding immune cells and triggering a pyroptotic chain reaction (23). Thus, pyroptosis plays a key role in the emergence of a cytokine storm, according to recent research (17, 19, 24).

Although pyroptosis is crucial for innate immunity (25, 26), extensive pyroptosis can cause tissue inflammation, organ failure and death, as found in various diseases (27). For example, in atherosclerosis, cholesterol crystals and oxidized low-density lipoprotein cause macrophage pyroptosis, which leads to a massive release of cytokines, promoting inflammation and disease progression (28). In line with these findings, NLRP3 inflammasome or ASC inhibition, which prevent macrophage pyroptosis, can lower infarct size and improve heart function in an animal model of myocardial infarction (29, 30). Similar results have also been observed in alcoholic hepatitis (31), lupus erythematosus (32, 33), and even in the central nervous system (34).

In COVID-19 patients, various cells undergo pyroptosis, including leukocytes (monocytes, macrophages, mucosal-associated invariant T cells) and other type of cells (adipocytes, lung epithelial cells and endothelial cells) (16, 35–39). On the one hand, SARS-CoV-2 nucleocapsid can prevent Gasdermin D cleavage, thus reducing host pyroptosis and suppressing the immune response (40), in addition to inhibiting coronavirus infection by promoting non-classical secretion of β-interferon (41). On the other hand, SARS-COV-2 can stimulate macrophage GSDMD-mediated pyroptosis, which leads to the rapid release of pro-inflammatory cytokines and to a cytokine storm (17). In addition, the synergistic effect of TNF-α and IFN-γ can trigger GSDMD-mediated pyroptosis and promote a cytokine storm, thereby increasing mortality among COVID19 patients (42). However, in cats and dogs, deficiencies of the inflammasome and pyroptosis pathways (cats and tigers do not express AIM2 and NLRP1, and dogs do not express AIM2 and have a shorter form of NLRC4 than humans) may provide an evolutionary advantage against SARS-CoV-2 by reducing cytokine storm-induced host damage (43). Therefore, the role of pyroptosis in COVID-19 remains complex, requiring more comprehensive studies.

Based on transcriptome data of patients with or without COVID-19 available in public databases (GSE157103), we found that the leukocytes of ARDS patients with COVID-19 have considerably higher pyroptotic markers than patients without COVID-19. Moreover, at least two different patterns of pyroptosis occur in patients with COVID-19, one correlated with a poor prognosis and the other with a benign prognosis. These two pyroptosis patterns may be regulated by different upstream transcription factor networks, which could prove therapeutically valuable for drug development.

## Materials and Methods

### Obtaining RNA-seq Data from the GEO Dataset

From the GEO dataset, we retrieved RNA-seq data (GSE157103) of 126 ARDS patients, namely 100 COVID-19 patients and 26 non-COVID-19 patients, in addition to their clinical data, including gender, age, underlying disease status (diabetes), coagulation (D-dimer, ferritin, CRP, procalcitonin, fibrinogen), and hospital-free days post 45-day follow-up (HFD45), among other parameters. More specifically, HFD45 is defined as the number of days patients lived outside of a hospital from enrollment through death or the end of follow-up (44). The higher HDF45 is, the milder the disease and the better the prognosis will be.

### Proteomic Data Collection from the MassIVE Database and Analysis

The label-free quantification (LFQ) intensities of 736 proteins of 126 ARDS patients were collected from Mass Spectrometry Interactive Virtual Environment (MassIVE) (MSV000085703). After calculating the logarithm of the FQL intensities, we used the R package “limma” to calculate the log2(Log2Fold of change) (log2FC) of 736 proteins between two PYRclusters. The 736 proteins were sorted from large to small by log2FC (not absolute value). Then, using the “org.Hs.eg.db” and “clusterProfiler” packages, gene set enrichment analysis (GSEA) was performed based on MSigDB gene sets C2, C5 and C7. Significant gene sets were identified when |Normalized Enrichment Score (NES)|>1 and False Discovery Rate (FDR) < 0.25.

### Function and Pathway Analysis of DE Immune Genes

Gene ontology (GO) and Kyoto Encyclopedia of Genes and Genomes (KEGG) analyses were performed using the “org.Hs.eg.db” and “clusterProfiler” packages. GO terms and KEGG terms were identified as significantly enriched when p.adjust < 0.05.

### Estimation of Immune Cell Infiltration Fractions

Single-sample gene set enrichment analysis (ssGSEA) and “Cibersort” were used to analyze the immune cell infiltration fractions. The former was based on the list of Pan-cancer Immune Metagenes (45, 46).

### Unsupervised Clustering of COVID-19 Patients

Based on RNA-seq data of 35 pyroptosis-related genes including *AIM2*, *CASP1*, *CASP3*, *CASP4*, *CASP5*, *CASP6*, *CASP8*, *CASP9*, *ELANE*, *GPX4*, *GSDMA*, *GSDMB*, *GSDMC*, *GSDMD*, *GSDME*, *GZMA*, *GZMB*, *IL18*, *IL1B*, *IL6*, *NLRC4*, *NLRP1*, *NLRP2*, *NLRP3*, *NLRP6*, *NLRP7*, *NOD1*, *NOD2*, *PJVK*, *PLCG1*, *PRKACA*, *PYCARD*, *SCAF11*, *TIRAP*, and *TNF*, we divided 100 COVID-19 patients into two clusters (PYRcluster) using the “nmf” package. We determine the k value based on the consensusMap function.

### Construction of the PYRscore

The differentially expressed genes (DEGs) of two PYRclusters were identified using the “limma” package. Using the median of HFD45 as the cutoff value, COVID-19 patients were divided into two groups. The DEGs in these two groups were calculated using the limma package and subsequently applied for PCA analysis. PC1 and PC2 were used to construct the PYRscore (47).


$$\text{PYRscore}=\sum \left(\text{PC}1_{\text{i}}+\text{PC}2_{\text{i}}\right)$$

### Construction of the PYRsafescore Model

Based on the log2(TPM) of the 570 DEGs between PYRclusters and the HDF45 of each patient, we used the “glmnet” package to build a PYRsafescore model by LASSO regression. We determine the signatures of the model by selecting the lambda value with the smallest mean-squared error by 20-fold-cross-validation. The coefficients of the final signatures were used to calculate the PYRsafescore as follows: protective score = ∑ Coefficient_i_ × Expression level of signature_i_. Using the “caret” package, 100 patients were randomly divided into a training group and a test group with a ratio of 2:1. The model built with the training group data was validated in the test group. We used “ROCR” packages to plot receiver operating characteristic (ROC) curves and to calculate area under the curve (AUC) scores to evaluate model performance.

### Transcription Factors Enrichment

First, based on the transcription factor targets (TFT) and their gene sets in MSigDB (48), we used the “clusterProfiler” package to enrich the transcription factors from DEGs between PYRclusters.

We then used the “CoRegNet” package to enrich the transcription factor co-regulatory network in COVID-19 patients from PYRcluster1 and PYRcluster2 based on the dataset of transcription factor targets (CHEA, ENCODE, and JASPAR Predicted Transcription Factor Targets, MotifMap and TRANSFAC Predicted Transcription Factor Targets, and TRANSFAC Curated Transcription Factor Targets) from Harmonizome (49, 50). Lastly, the network graph was plotted using the “ggraph” package.

### Search for Drugs Targeting Transcription Factors

We used the transcription factors that we screened as keywords to search for the corresponding compounds on ChEMBL (51), thus screening active or repressed compounds based on their role in pyroptosis.

### Statistical analysis

The Wilcoxon sum-rank test and the t-test were used to compare different groups, and the Pearson’s product-moment correlation test was used for correlation analysis. All statistical tests were two-sided, and a significant difference was defined as a p-value of 0.05. Power calculations were performed using the following R packages: “pwr” and “rstatix” at sig.level=0.05.

## Results

### Transcriptome Data Reveal the Pyroptosis Characteristic of Blood Leukocytes from COVID-19 Patients

A GEO dataset provided RNA-seq data and clinical data from 126 samples of 100 patients with COVID-19 and 26 patients without COVID-19 (GSE157103) (44). Initially, we assessed the expression levels of pyroptosis-related gene sets of all patients based on prior studies (52).

**Figure 1:**
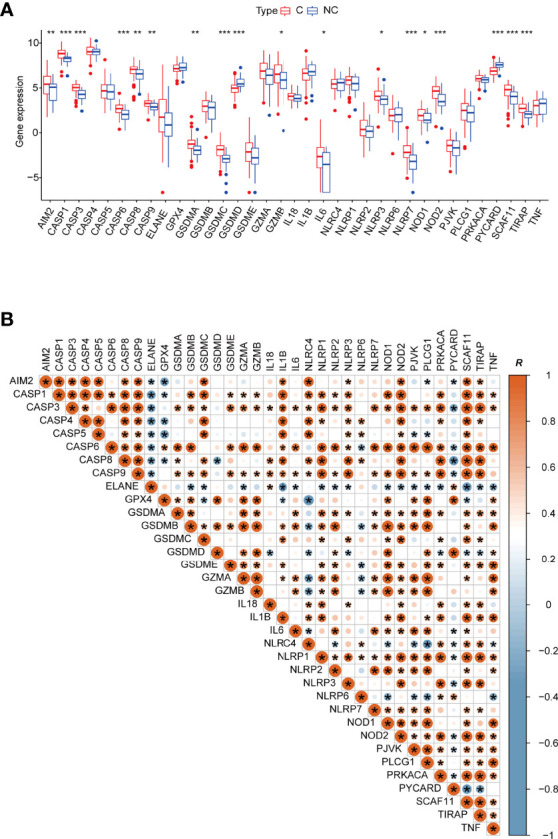
**(A)** Boxplot of 35 pyroptosis-related genes’ relative expression between different types of patients. C: COVID-19 patients; NC: none-COVID-19 patients. **(B)** The Pearson’s correlation between 35 pyroptosis-related genes in COVID -19 patients, R value represents the Pearson’s correlation coefficient. *p < 0.05, **p < 0.01, ***p < 0.001, ****p < 0.0001.

The expression of the pyroptosis-related genes *AIM2*, *CASP1*, *CASP3*, *CASP6*, *CASP8*, *CASP9*, *GSDMA*, *GSDMC*, *GZMB*, *IL6*, *NLRP3*, *NLRP7*, *NOD1*, *NOD2*, *SCAF11*, and *TIRAP* was significantly higher in blood leukocytes of COVID-19 patients, indicating that the level of pyroptosis was significantly increased (**Figure 1A**). *NLRP3*, *NLRP7*, *NOD1*, *NOD2* are closely related to caspase activation (53, 54).

Subsequently, we outlined the correlation patterns of 35 pyroptosis-related genes in COVID-19 patients to investigate relationships between different pyroptosis-related genes. Although most of the 35 genes have a substantial positive association with other genes, several genes are nevertheless negatively correlated with other pyroptosis-related genes. *ELANE*, for example, is negatively correlated with *AIM2*, *CASP1*, *CASP3*, *CASP4*, *CASP5*, *CASP6*, *CASP8*, *CASP9*, *GSDMB*, *GSDMC*, *GZMA*, *IL1B*, *IL6*, *NLRP1*, *NLRP2*, *NLRP3*, *NOD1*, *NOD2*, *PJVK*, *PLCG1*, *PRKACA*, *SCAF11*, *TIRAP*, and *TNF*. Therefore, the expression of these pyroptosis-related genes in COVID-19 patients presents complex patterns (**Figure 1B**). Furthermore, each caspase is strongly and positively correlated with the others. For example, the linkage between GSDMA and caspases suggests that its cleavage is related to caspase-3/-6/-8/-9. CASP3 expression is highly linked to GSDME, in line with previous reports on caspase-3 cleavage of GSDME, releasing its activity (22).

**Figure 2:**
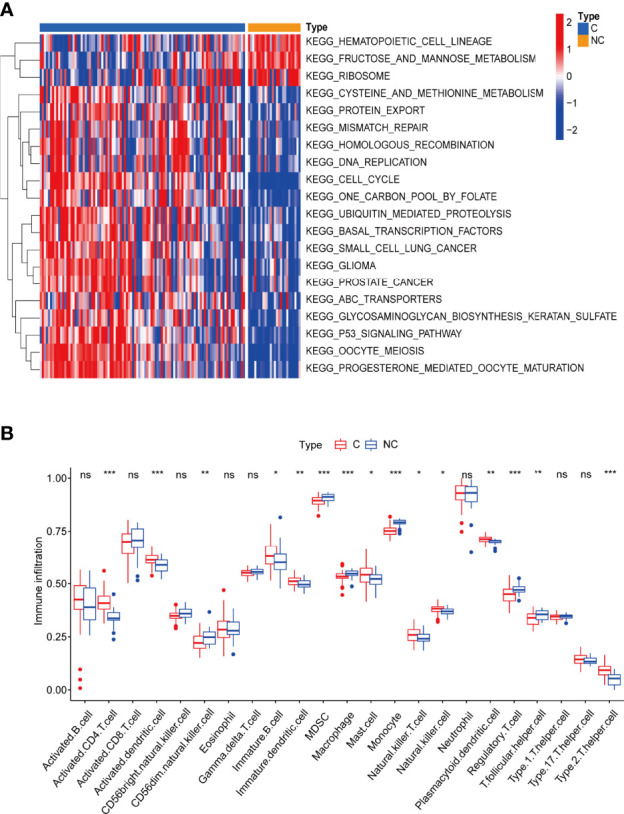
**(A)** Gene set variation analysis (GVSA) analysis shows COVID-19 patients’ leukocytes may have been significantly damaged during viral infection and are undergoing damage repair. C: COVID-19 patients; NC: none-COVID-19 patients. **(B)** The abundance of leukocytes between the different types of patients. *p < 0.05, **p < 0.01, ***p < 0.001, ****p < 0.0001, ns, no significance.

By gene set variation analysis (GSVA), we studied changes in the biological function of leukocytes between the two types of patients. Mismatch repair, homologous recombination, replication, cell cycle, and p53 signaling are more enriched in leukocytes of COVID-19 patients, indicating severe cell damage during viral infection and ongoing damage repair (**Figure 2A** and **Supplementary Table 1**).

By single-sample gene set enrichment analysis (ssGSEA), we also compared the proportions of 28 immune cell types between the two groups and found that the numbers of various immune cells are much higher in COVID-19 patients than in non-COVID-19 patients, indicating a highly active immune response in COVID-19 patients (46) (**Figure 2B** and **Supplementary Table 2**). Surprisingly, the numbers of some immune cells, such as macrophage and cd56^dim^ natural killer cells, were lower in COVID-19 patients than in the control group. This difference could be due to cell death caused by massive viral infection and increased pyroptosis (17, 55). Human dendritic cells and T lymphocytes (including CD4^+^ and CD8^+^) undergo pyroptosis *via* the AIM2-Caspase1-gasdermin D and the CARD8-Caspase1-gasdermin D axes (56, 57), respectively. Pyroptosis has also been identified in macrophages and neutrophils (58, 59). Although NK cells have not been documented to undergo pyroptosis on their own, they can participate in this process, playing a key role (60). These results suggests that blood leukocytes either directly undergo pyroptosis or have a synergistic role with pyroptosis in COVID-19 patients.

In summary, blood leukocytes exhibit substantial pyroptotic characteristics in COVID-19 patients.

### COVID-19 Patients Showed Different Patterns of Pyroptosis

**Figure 3:**
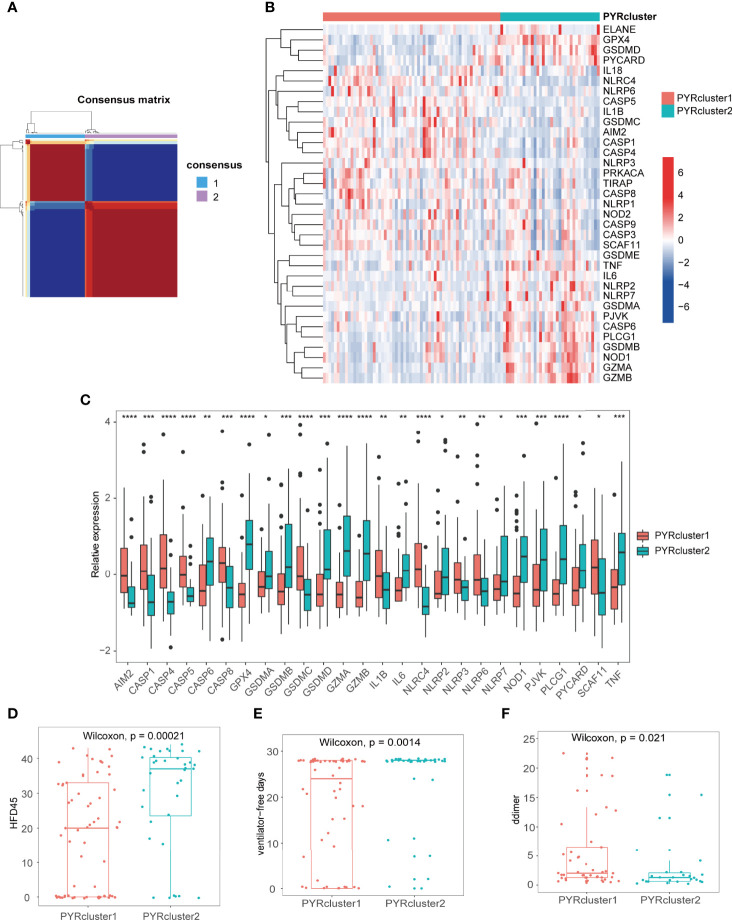
**(A)** Consensus clustering matrix for k = 2. **(B)** The heatmap of 35 pyroptosis-related genes between the two PYRclusters. Red represents high expression; blue represents low expression. **(C)** Boxplot of significant pyroptosis-related genes’ relative expression between two PYRclusters. **(D–F)** The HFD45, ventilator-free days, D-dimer levels between the two PYRclusters. *p < 0.05, **p < 0.01, ***p < 0.001, ****p < 0.0001, ns, no significance.

By non-negative matrix factorization based on 35 pyroptosis-related genes, we clustered the 100 COVID-19 patients into two clusters, PYRcluster1 and PYRcluster2 (**Figure 3A** and **Supplementary Figure 1A**). The best clustering result was found when k = 2, with no differences in age, gender, days admitted before enrollment, replacement therapy (pre-enrollment) or underlying disorders between the two clusters, which we called PYRclusters (**Supplementary Figures 1B–E**, **3A, B**).

We first explored differences in the expression of pyroptosis-related genes between the two PYRclusters (**Figures 3B,C** and **Supplementary Table 3**) and found that PYRcluster1 has a higher expression of *NLRC4*, *NLRP6*, *CASP5*, *CASP8*, *CASP9*, *GSDMC*, and *AIM2*, whereas PYRcluster2 has a higher expression of *GPX4*, *GSDMD*, *PYCARD*, *TNF*, *IL6*, *NLRP2*, *NLRP7*, *GSDMA*, *CASP6*, *GSDMB*, *NOD1*, *GZMA*, and *GZMB*.

Subsequently, we found that PYRcluster2 has a higher hospital-free days post 45-day follow-up (HFD45), ventilator-free days and a lower proportion of mechanical ventilation than PYRcluster1 (**Figures 3D, E** and **Supplementary Figure 3C**), which suggests a better prognosis. The blood D-dimer level of PYRcluster1 is significantly higher than that of PYRcluster2 (**Figure 3F**), indicating hypercoagulation. Although albumin and hemoglobin of the two PYRclusters are mostly below the normal range (green dashed line), PYRcluster1 deviates further from the normal range than PYRcluster2. Other clinical features do not differ significantly between the two clusters (**Supplementary Figures 2A–C**). Based on these results, the highly expressed pyroptosis-related genes of PYRcluster1 may be associated with a poor prognosis. In line with our results, *AIM2* and *NLRC4* deficiency in dogs and cats provide a protective effect against SARS-CoV-2 by reducing cytokine storm-induced host damage (43).

**Figure 4:**
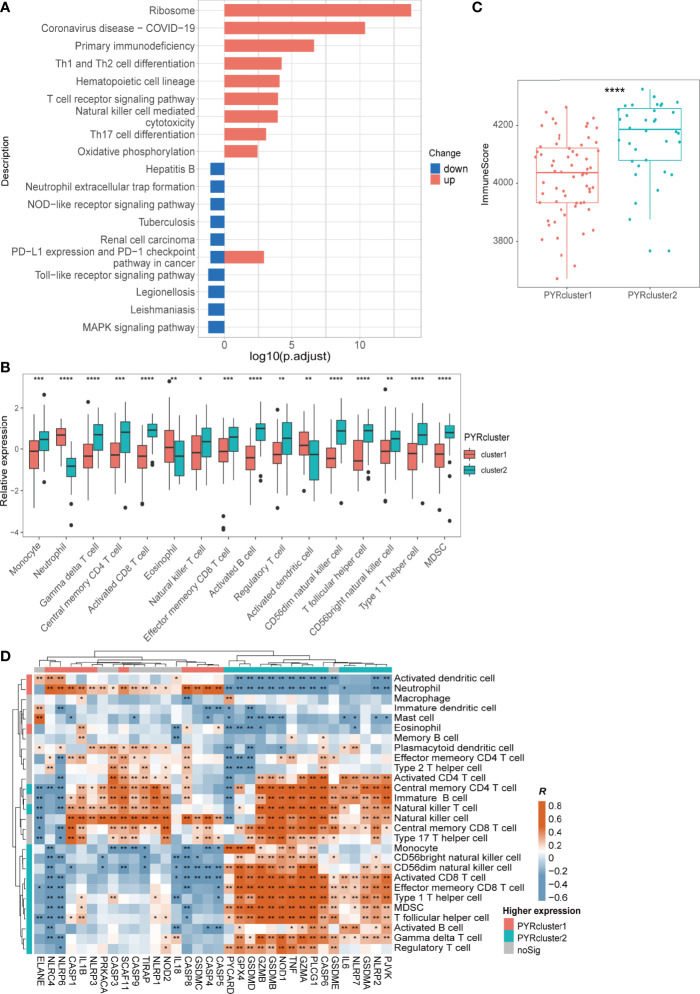
**(A)** Kyoto Encyclopedia of Genes and Genomes (KEGG) enrichment of 570 DEGs Between two PYRclusters, “up” means these pathways of PYRcluster2 were upregulated when compared to PYRcluster1; “down” means these pathways were downregulated. **(B)** Leukocytes with significantly different expression levels among PYRclusters. **(C)** ImmuneScore calculated by “estimate” package between two PYRclusters. **(D)** Pearson’s correlation between expressions of 35 pyroptosis-related genes and abundance of leukocytes, R value represents the Pearson’s correlation coefficient. Annotated bars above and to the left indicate in which PYRcluster each pyroptosis-related gene or leukocyte is highly expressed.

We further determined differentially expressed genes (DEGs) using the “limma” package, with cutoff criteria of |logFC| >1 and *p*=0.05, totaling 570 DEGs, and employed Gene ontology (GO), Kyoto Encyclopedia of Genes and Genomes (KEGG) pathway enrichments (**Figure 4A** and **Supplementary Figure 2E**). In addition, we determined the logFC of 736 proteins between two PYRclusters (FC=PYRcluster2/PYRcluster1) using “limma” from the proteomic data from the same study as the transcriptome data. Subsequently, we employed gene set enrichment analysis (GSEA) based on the log2FC of these proteins (**Supplementary Figure 2D**, **Supplementary Table 4**).

Immune responses such as antigen recognition/presentation, immune cell activation, migration, and replication are relatively enhanced in PYRcluster2 (**Figure 4A** and **Supplementary Figure 2E**). Both transcriptome and proteomic results revealed hyperinflammation in PYRcluster1 (**Supplementary Figure 2D**, **Supplementary Table 6**). KEGG pathway enrichment data showed that “Coronavirus disease-COVID-19” pathways are markedly upregulated in PYRcluster2, underscoring a highly activated immune process unique to PYRcluster2. PYRcluster1 has a higher NO synthesis level than PYRcluster2, which may be related to the antiviral capacity of its patients (61–63). The highly activated stress response pathway may associated with severe damage caused by the virus and lead to higher levels of immune cell apoptosis in PYRcluster1 (**Supplementary Figure 2D**, **Supplementary Table 6**). Furthermore, PYRcluster1 has a markedly increased expression of cytokines, including IL-1, IL-6, IL-8, IL-10, and TNF (**Supplementary Table 6**), which are typical components of a cytokine storm and result in a poor prognosis (64, 65). Consistent with an increased D-dimer (**Figure 3F**), the neutrophil extracellular traps (NETs) that promote blood coagulation are highly expressed in PYRcluster1, promoting venous thrombosis and leading to poor prognosis (66, 67). In addition, *AIM2*, *CXCR2* and *UBE2W*, which were significantly highly expressed in PYRcluster1, were included in the 20 genes associated with distinctly methylated CpG sites between mild and severe COVID-19 patients (68). Their odds ratios were all greater than 1, indicating that their downregulation is beneficial to COVID-19 patients. In conclusion, the two PYRclusters of COVID-19 patients exhibited distinct pyroptotic patterns and clinical features.

### Different Pyroptotic Patterns are Associated with Different Leukocytes and Opposite Prognosis

SARS-CoV-2 viruses infect leukocytes and lead to immunodeficiency (69–71). We speculate that leukocyte pyroptosis helps to destroy the virus protective niche and release viruses from cells, thereby enhancing viral clearance and immune recovery. As a result, viruses released by pyroptosis may be further removed by phagocytic cells *via* phagocytosis.

We first assessed the proportion of different immune cells in the two PYRclusters by ssGSEA. Immune cells highly associated with antivirals, such as activated B, CD4/8^+^ T, Treg, and NK cells are highly expressed in PYRcluster2 (**Figure 4B** and **Supplementary Figure 4A**, **Supplementary Table 7**). PYRcluster1 had a higher proportion of pro-inflammatory neutrophils. Using “Cibersort”, we discovered PYRcluster2 had a higher proportion of anti-inflammatory M2 macrophages (**Supplementary Figure 4A**). The hemogram percentages of several cells, such as neutrophils, lymphocytes and monocytes, were consistent with the results of ssGSEA (**Supplementary Figure 3F**). We then used the “estimate” package to score the two clusters and found that PYRcluster2 shows a greater increase in immune cell infiltration than PYRcluster1 (**Figure 4C**). As shown in **Figure 4D**, the expression of characteristic pyroptosis-related genes of PYRcluster1 were significantly positively correlated with its expression of characteristic immune cells. For example, *PYCARD*, *GPX4*, *GSDMD*, *GZMA*, *TNF*, *NOD1*, *IL6*, *NLRP7*, *CASP6*, *GSDME*, *PJVK*, *GSDMA*, and *NLPR2* are positively correlated with NK, NKT, regulatory T, γδ T, activated B, and Th17 cells, MDSCs, and monocytes (**Figure 4D**). Both these pyroptosis-related genes and immune cells are highly expressed in PYRcluster2 (**Figures 3C**, **4B**). Furthermore, leukocytes have stronger phagocytic activity in GO enrichment results in PYRcluster2 than in PYRcluster1 (**Supplementary Table 6**). In contrast, pyroptosis in PYRcluster1 may produce several pathogen- and damage-related molecular patterns that increase cytokine storm, leading to multiple organ failure and poor prognosis.

**Figure 5:**
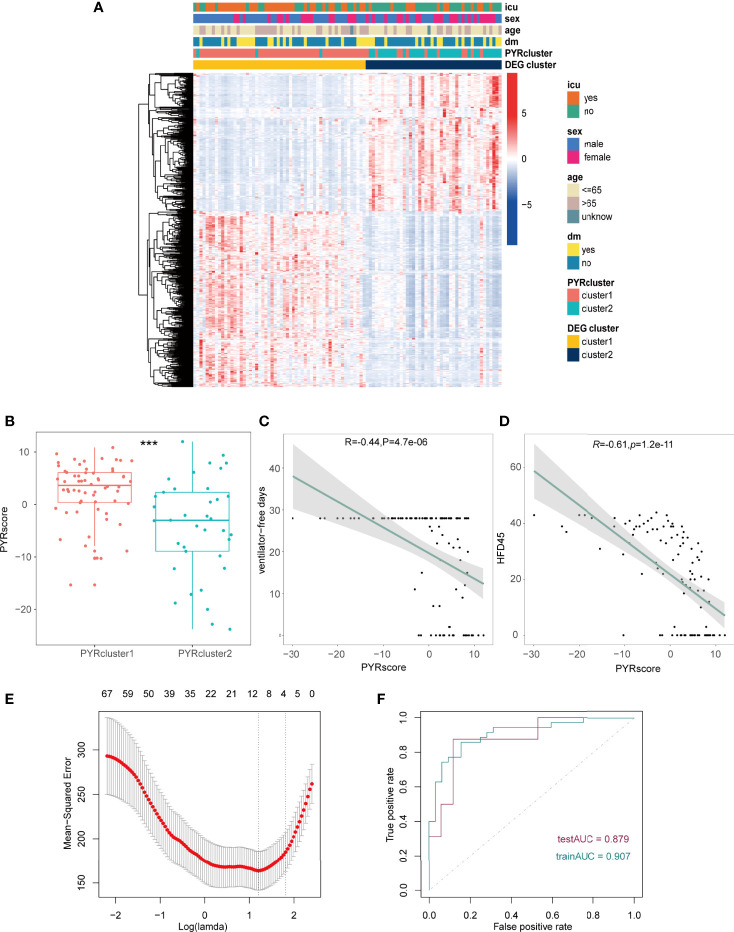
**(A)** Heatmap of the DEGs between the gene clusters, different clinical data was shown in the annotation. **(B)** Pyrscore between two PYRclusters. **(C, D)** Pearson’s correlations between pyrscore and ventilator-free days**(C)**, HFD45 **(D)**, R value represents the Pearson’s correlation coefficient; grey area represents the 95% confidence interval for the linear fit. The maximum value of ventilator-free days is 28 since this 28-day time frame was initially chosen because most subjects with ARDS will have died or been extubated by Day 28. **(E)** Mean-squared error (MSE) of different numbers of variables revealed by the LASSO regression model. The red dots represent the MSE values; the grey lines represent the standard error (SE); the two vertical dotted lines on the left and right, respectively, represent optimal values by minimum criteria and 1-SE criteria. “Lambda” is the tuning parameter. **(F)** AUC of patients in the training group and test group. *p < 0.05, **p < 0.01, ***p < 0.001, ****p < 0.0001, ns, no significance.

We subsequently performed unsupervised clustering of all COVID-19 patients using 570 DEGs and all genes, respectively, yielding two more clusters: DEG and All-Gene clusters. The heatmap demonstrates that these additional clusters match a previous clustering based on the 35 pyroptosis-related genes (**Figure 5A** and **Supplementary Figure 4B–D**). These data suggest that distinct patterns of pyroptosis occur in COVID-19 patients, which can be represented by 35 pyroptosis-related genes.

To better elucidate differences in pyroptotic patterns between PYRcluster1 and PYRcluster2 and their correlation with prognosis, we created a pyroptosis score (pyrscore) (**Supplementary Figure 4E**). As shown in **Figure 5B**, PYRcluster1 has a higher score than PYRcluster2. HFD45 and ventilator-free days are negatively correlated with pyroptosis scores (**Figures 5C, D**), whereas sofa, APACHE-II, D-dimer, and CRP levels are positively correlated (**Supplementary Figures 5A–D**). In conclusion, PYRcluster1 and PYRcluster2 have different levels of immune response to SARS-COV-2 and pyroptotic patterns, resulting in distinct prognoses.

### Development of A Predictive Pyroptotic Prognosis Model

Given that different pyroptotic patterns may have a significant impact on the prognosis of COVID-19 patients, we created a PYRsafescore model based on the HFD45 of COVID-19 patients and DEGs across PYRclusters by least absolute shrinkage and selection operator (LASSO) regression analysis. In a ratio of 2:1, 100 COVID-19 patients were divided into a training group and a test group, and the model was obtained in the training group. “Lambda-min” was chosen as the best value in the cross-validation procedure (**Figure 5E** and **Supplementary Figures 5E**). Lastly, based on the log2 value of the expression level of 10 genes, we established the following scoring model:


$$\begin{array}{l} PYRsafescore=0.024522643\times PEBP1+0.279568268\times IL2RB+3.19881976\times HLA-DMB+\\ 0.477980193\times ZNF683+\left(-0.553440043\;\times IL1R2\right)+\left(-0.318430005\;\times C3orf86\right)+1.999292597\times \\ CD8A+0.82422511\times TGFBI+\left(-0.050606043\times ADAMTS2\right)+0.29910421\times FCER1A. \end{array}$$

**Figure 6:**
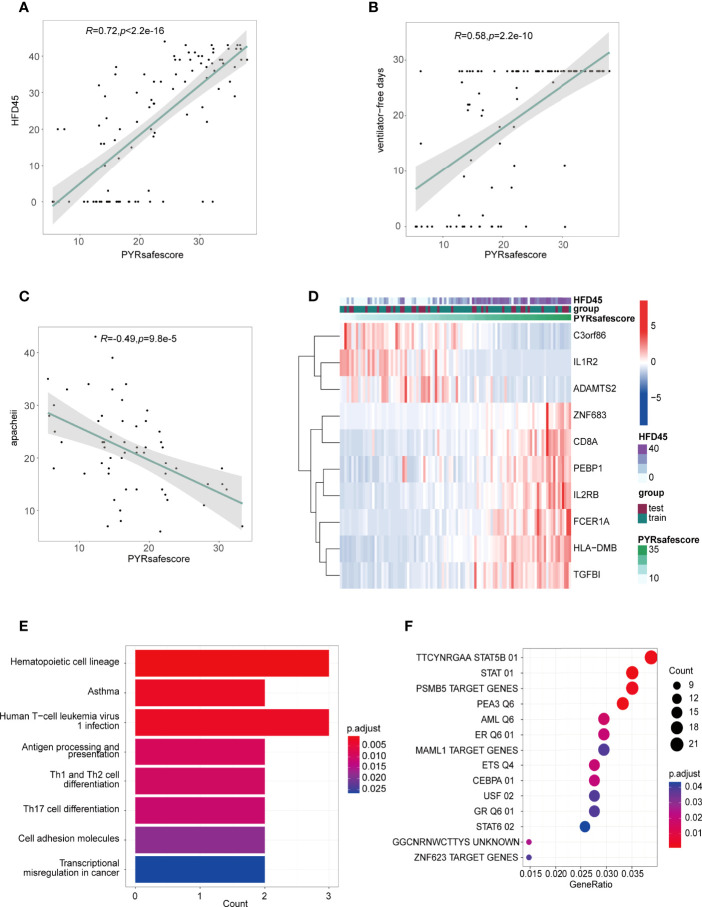
**(A, B, C)** Pearson’s correlations between PYRsafescore and HFD45 **(A)**, ventilator-free days **(B)**, APACHE-II **(C)**, R value represents the Pearson’s correlation coefficient; grey area represents the 95% confidence interval for the linear fit. **(D)** Heatmap of signature genes of PYRsafescore; expression of these genes was highly correlated with HFD45 and PYRsafescore. **(E)** Kyoto Encyclopedia of Genes and Genomes (KEGG) enrichment of signature genes of PYRsafescore. **(F)** Transcription factor enrichment of 570 DEGs between PYRclusters using “clusterProfiler” package based on MSigDB gene set: TFT (transcription factor targets) gene set.

The area under the ROC curve (AUC) values of the model in the training and test groups were 0.907 and 0.879, respectively, indicating that our predictive model performs well (**Figure 5F**). HFD45 and PYRsafescore are positively correlated (**Figure 6A**), which suggests that a higher PYRsafescore indicates a better prognosis. In addition to HDF45, the correlation of other clinical variables with PYRsafescore, including sofa, ventilator-free days, APACHE-II, and CRP levels, also demonstrates that our model works effectively (**Figures 6B, C** and **Supplementary Figures 6A, B**). Additionally, the expression of these 10 genes between the two PYRclusters also has significant differences (**Figure 6D**). KEGG analysis indicates that they were closely related to the patient’s immune response (**Figure 6E**).

In conclusion, our results proved that the newly created prognosis model has a considerable clinical predictive value.

### Transcriptional Regulatory Networks and Potential Drugs in Different Pyroptosis Patterns

**Figure 7:**
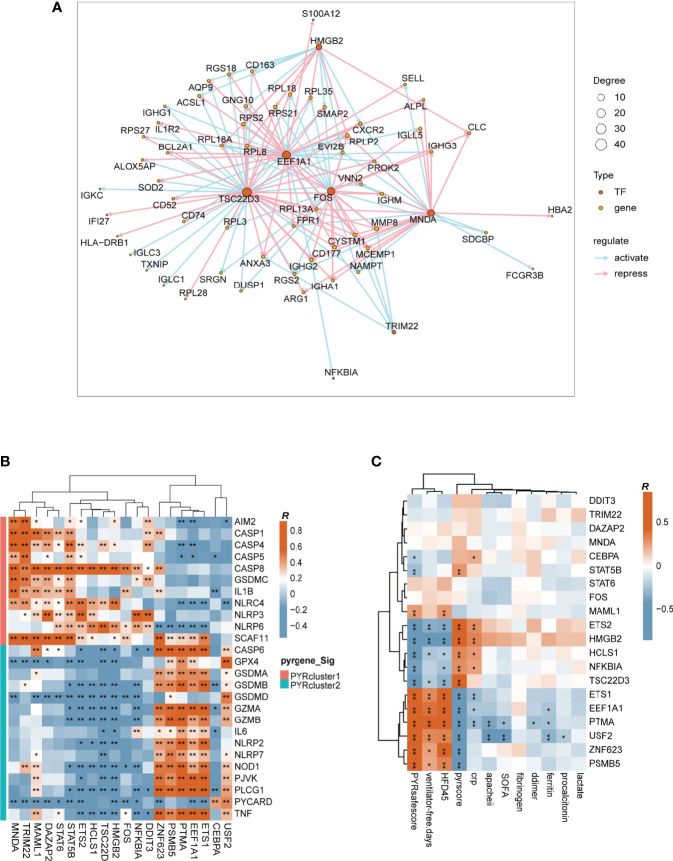
**(A)** Transcription factors regulatory network of PYRcluster1. “Degree” means the number of edges connected to the node. **(B)** Pearson’s correlation of differentially expressed pyroptosis-related genes and transcription factors in PYRclusters; R value represents the Pearson’s correlation coefficient. Annotation on the left represents in which PYRcluster each pyroptosis-related gene is significantly highly expressed. **(C)** Pearson’s correlation between different clinical data and transcription factors; R value represents the Pearson’s correlation coefficient.

Using the “clusterProfiler” package, we first enriched DEGs across PYRclusters for transcription factors based on MSigDB Collections: “regulatory target gene sets” (**Figure 6F**). We further enriched the transcription factor regulatory networks for all transcriptome data of the two PYRclusters using the “CoRegNet” package. PYRcluster1 has the regulatory network with *MNDA, TSC22D3, HMGB2, FOS, EEF1A1, TRIM22, NFKBIA* as transcription factors, and PYRcluster2 has the regulatory network with *FOS, MNDA, EEF1A1, DAZAP2, DDIT3, HCLS1, NFKBIA, TSC22D3, PTMA*, and *TRIM22* (**Figure 7A** and **Supplementary Figure 6C**, **Supplementary Table 8**). Notably, both PYRcluster1 and PYRcluster2 are regulated by *FOS*, *EEF1A1*, *MNDA*, and *TRIM22*.

Once SARS-COV-2 infects cells, EEF1A1 is critical for viral replication. Drug targeting EEF1A has robust antiviral effects *in vitro* (72). The nuclear factor-kappaB (NF-kappaB)/REL family of transcription factors are activated in response to DNA damage to regulate inflammation and apoptosis resistance (73, 74). Since NFKBIA is highly expressed in COVID-19 patients (75) and tripartite motif-containing (TRIM) 22 can activate NF-kappaB (76) to protect the host from viral infection (77), these two genes have a huge impact on immune disparities between the two PYRclusters. MNDA is a member of the family of hematopoietic interferon (IFN)-inducible nuclear proteins that promotes the degradation of the anti-apoptotic factor MCL-1 and apoptosis in myeloid cells (78, 79).

We then observed the correlation between these transcription factors and the expression of differentially expressed pyroptosis-related genes between PYRclusters and the clinical characteristics of all patients. The correlations between transcription factors and pyroptosis-related genes vary greatly, depending on the PYRclusters (**Figure 7B**). Pyroptosis-related genes highly expressed in PYRcluster1 are positively correlated with *FOS*, *MAML1*, *DAZAP2*, *STAT6*, *STAT5B*, *ETS2*, *HCLS1*, *TSC22D3*, *HMGB2*, *MNDA*, *TRIM22*, *NFKBIA*, and *DDIT3*, whereas genes highly expressed in PYRcluster2 are positively correlated with *ZNF623*, *PSMB5*, *PTMA*, *EEF1A1*, *ETS1*, *CEBPA*, and *USF2*. Moreover, some transcription factors positively correlated with pyroptosis-related genes in PYRcluster2 also have a positive effect on prognosis (**Figure 7C**).

Taken together, these findings show that distinct pyroptotic patterns may result from different upstream transcriptional regulation pathways. In light of this, we searched the ChEMBL database for compounds that promote or inhibit appropriate transcription factors based on diverse roles in prognosis (51). We screened a total of 112 compounds (**Supplementary Table 9**) and found that CHEMBL348436 (also known as Cirsimaritin) has the potential to regulate blood leukocyte pyroptosis in COVID-19 patients while simultaneously improving prognosis through FOS and NFKB1A inhibition. In fact, drugs targeting FOS have therapeutic effects in COVID-19 patients (80).

## Discussion

Pyroptosis, a mechanism of programed cell death which leads to cell swelling and lysis, plays a key role in innate immunity by disrupting the pathogen replication niche and killing intracellular bacteria through pore-induced intracellular traps (25, 26). However, excessive pyroptosis may trigger an overactive inflammatory response, resulting in a cytokine storm and severe organ damage through IL-6, TNF and NETs (81–83).

The occurrence of a cytokine storm is a major factor in the progression of moderate-to-severe COVID-19. In the therapy of COVID-19, multi-organ failure induced by cytokine storm has become a significant issue (84). Despite the relevance of pyroptosis in the treatment of severe COVID-19 patients, research on COVID-19 and pyroptosis is currently limited. Some studies suggest that the elevated pyroptosis is not conducive to the treatment of the disease but closely related to SARS-CoV-2 infection and cytokine storm (17, 35, 42, 43). By contrast, other studies show that pyroptosis can also be beneficial in fighting SARS-CoV-2 infection (40, 41). A dual role for NLRP3 was reported in a recent study according to which inflammasome-dependent pyroptosis contributes to the hyperinflammatory state of the lungs. However, pyroptosis can release infectious virus, preventing a productive viral cycle, which can help to eliminate viruses (85). In conclusion, the role of pyroptosis in COVID-19 remains unclear.

From the transcriptome data of COVID-19 patients, we found that the blood leukocytes of COVID-19 patients have typical characteristics of pyroptosis. Of the 35 pyroptosis-related genes retrieved from the database, 19 are significantly elevated in COVID-19 patients. Using an unsupervised clustering approach with non-negative matrix factorization, we classified the COVID-19 patients into two populations with distinct pyroptosis patterns. PYRcluster1 featured high *AIM2*, *CASP1*, *CASP4*, *CASP5*, *CASP8*, *GSDMC*, *IL1B*, *NLRC4*, *NLRP3*, *NLRP6*, and *SCAF11* expression, whereas PYRcluster2 featured high *CASP6*, *GPX4*, *GSDMA*, *GSDMB*, *GSDMD*, *GZMA*, *GZMB*, *IL6*, *NLRP2*, *NLRP7*, *NOD1*, *PJVK*, *PLCG1*, *PYCARD*, and *TNF* expression.

Although the most well-known pyroptotic pathway in the present study contain *NLRP3*, *CASP1*, and *GSDMD* (21), pyroptosis can be induced by different inflammatory caspases and involves varied gasdermin proteins, such as *NLRC4*, caspase-3, caspase-8, caspase-11/4/5 and *GSDMC* (20, 86–89).

In combination with other clinical information, we found that PYRcluster2 patients had a better prognosis, including a longer HFD45 and a lower ICU hospitalization rate. PYRcluster2 patients also had more immune cells and a higher immune score. Furthermore, the expression of immune cells was highly correlated with the expression of pyroptosis-related genes. To better elucidate the pyroptotic patterns and prognosis, we calculated the “pyrscore” to characterize different pyroptotic patterns and the “PYRsafescore” to better predict prognosis and assist clinical treatment. Higher PYRsafescore scores mean a better prognosis.

Lastly, by transcription factor enrichment, we identified the upstream transcription factors that regulate different pyroptotic patterns and screened a series of compounds with therapeutic potential in public databases.

Currently, only a few drugs are available for controlling SARS-CoV-2 infection, including monoclonal antibodies that neutralize viral proteins (90–92), drugs that inhibit viral replication (93), and new oral drugs from Pfizer and Merck, namely PAXLOVID and Molnupiravir, which inhibit viral replication and viral proteases, respectively (94, 95). Methods for managing excessive inflammatory response, which is the leading cause of severe COVID-19, are very limited and less effective (96): For example, heparin is widely used to prevent blood clots, and some immunosuppressants such as dexamethasone, IL-6 monoclonal antibodies and JAK kinase family inhibitors are used to inhibit inflammation (12–15, 97–99). Our results suggest that pyroptosis plays a key role in the generation of a hyperinflammatory immune response. Therefore, therapeutic strategies targeting pyroptosis have potential value in managing inflammation and hence reducing COVID-19 severity and mortality.

**Figure 8:**
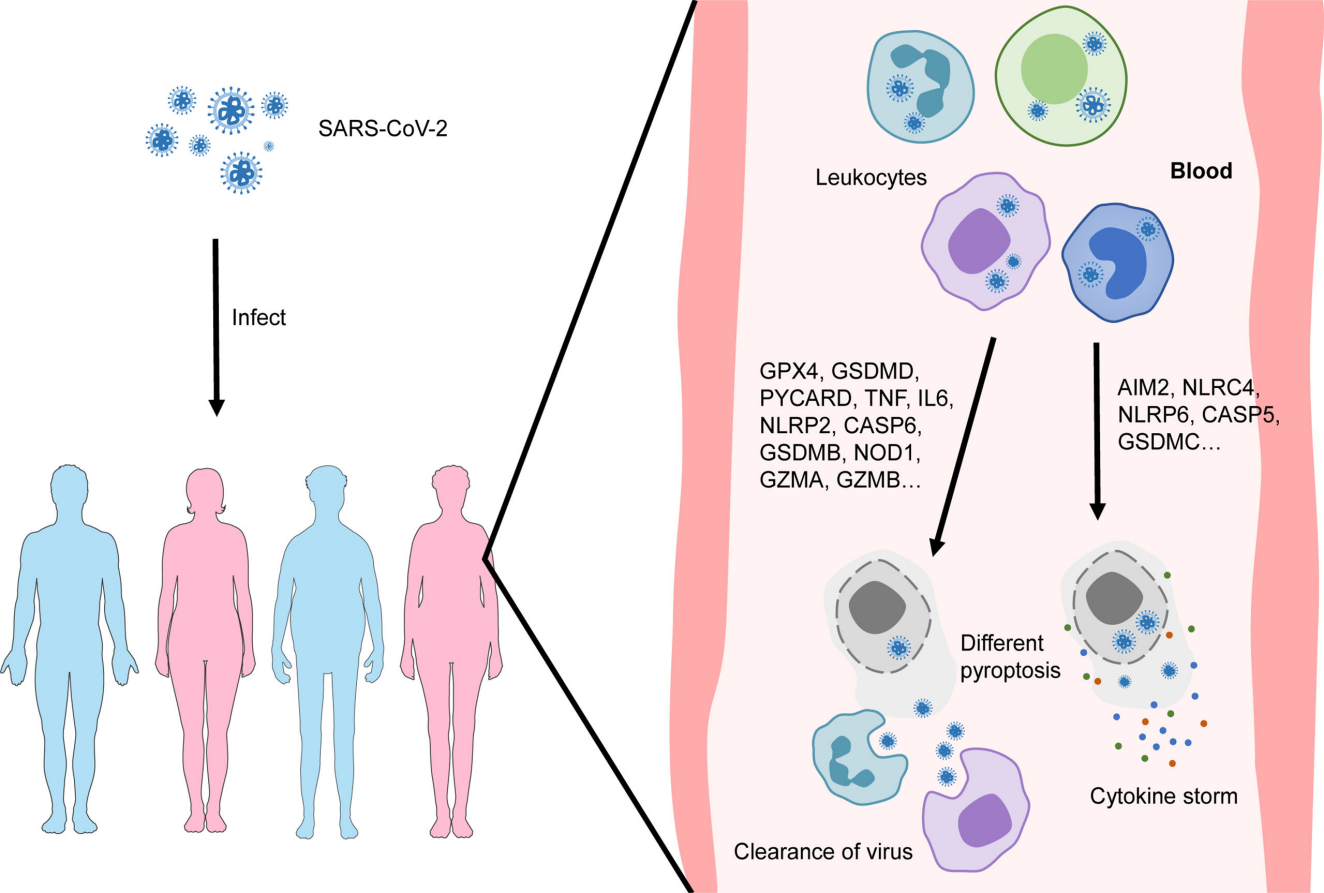
Different patterns of pyroptosis of blood leukocytes in patients with COVID-19.

In conclusion, our data reveal different patterns of pyroptosis of blood leukocytes in patients with COVID-19, which is closely related to their prognosis. We speculate the mechanism underlying diverse prognoses is shown in **Figure 8**. Prognosis prediction models developed based on different pyroptosis patterns are highly valuable for COVID-19 treatment. In addition, compounds that target the transcription factor network that regulates the pyroptotic process may help to develop new drugs for the treatment of patients with severe COVID-19.

## Data Availability Statement

The dataset GSE157103 used in this study is available from: National Center for Biotechnology Information (NCBI), Gene Expression Omnibus (GEO), https://www.ncbi.nlm.nih.gov/geo/query/acc.cgi?acc=GSE157103. The dataset MSV000085703 is available from: the MassIVE database (accession number MSV000085703; https://doi.org/10.25345/C5F74G).

## Author Contributions

YT, PZ, and LC designed and supervised the study, PZ collected the data, PZ performed the first part of the analysis, and YT completed the analysis and drafted the manuscript. YT, PZ, and QL prepared the original draft and wrote, reviewed, and edited the manuscript. JX and LC revised the manuscript and provided analytical technical support. All authors contributed to the article and approved the submitted version.

## Funding

This research was funded by the National Natural Science Foundation of China (32170750).

## Conflict of Interest

The authors declare that the research was conducted in the absence of any commercial or financial relationships that could be construed as a potential conflict of interest.

## Publisher’s Note

All claims expressed in this article are solely those of the authors and do not necessarily represent those of their affiliated organizations, or those of the publisher, the editors and the reviewers. Any product that may be evaluated in this article, or claim that may be made by its manufacturer, is not guaranteed or endorsed by the publisher.
