# Supplementary material for: Pyroptotic Patterns in Blood Leukocytes Predict Disease Severity and Outcome in COVID-19 Patients
Source: Front Immunol. 2022 Jul 19;13:888661. doi: 10.3389/fimmu.2022.888661 (PMC9343985; doi:10.3389/fimmu.2022.888661)
Supplement: Supplementary file 1 [file DataSheet_1.pdf]

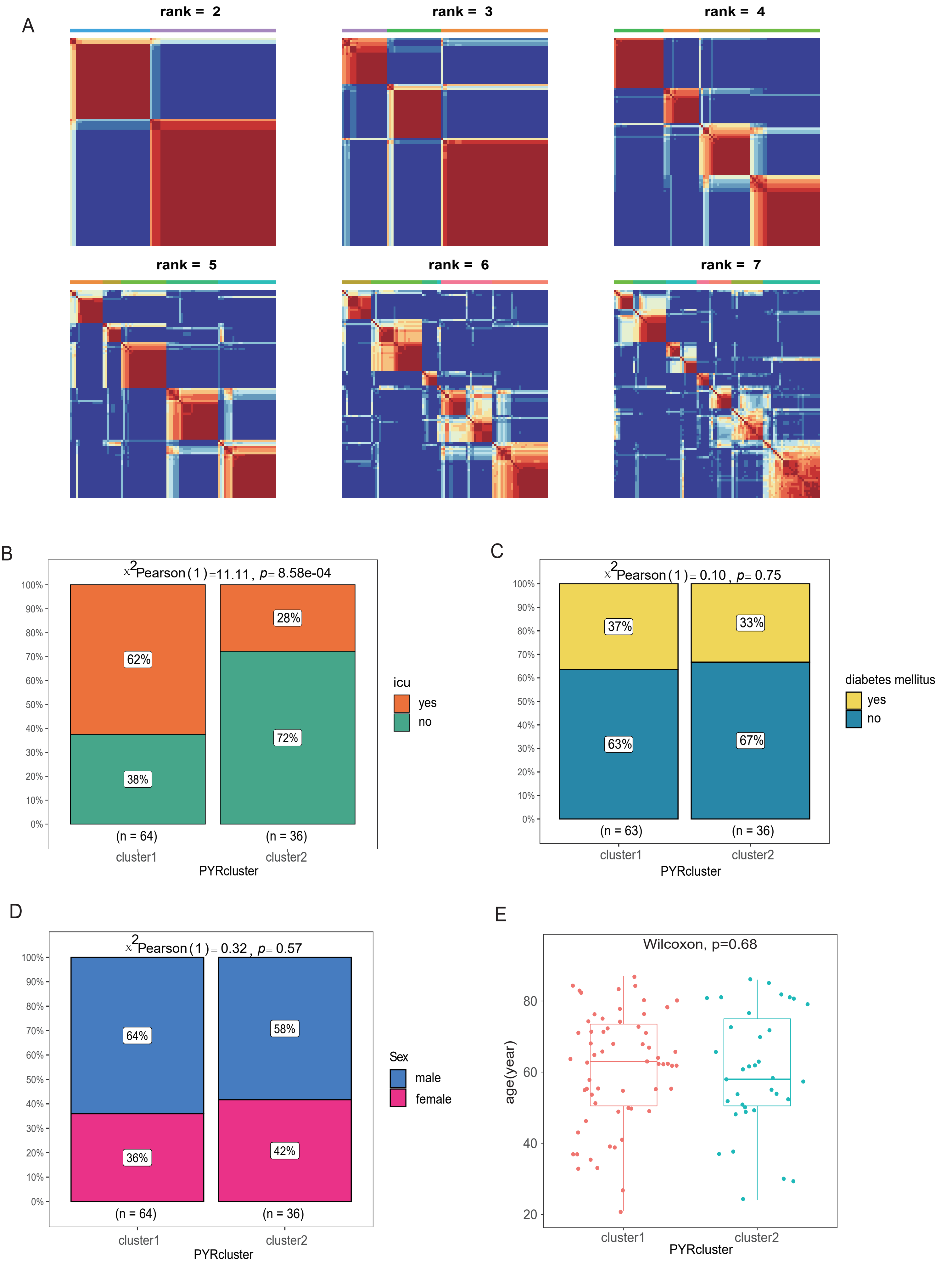

Figure 1: (A) Consensus clustering matrix for k = 2-7 revealed that clustering performance was optimized when k=2 (B, C, D, E), with no significant differences in the proportion of ICU, diabetes mellitus, sex, and age between the two PYRclusters.

Supplementary figure 1

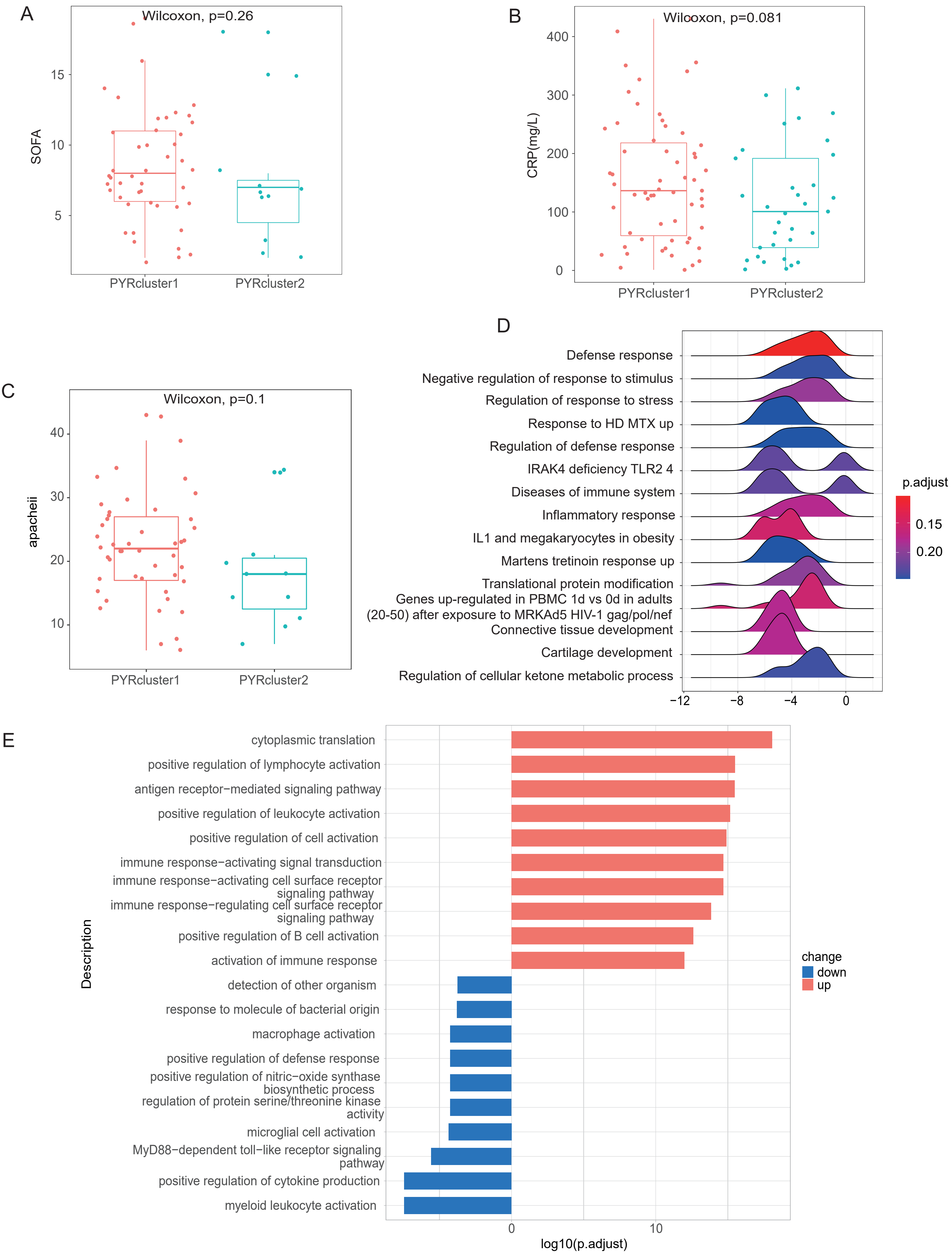

Figure 2: (A, B, C) SOFA, APACHE-II, and C-reaction protein (CRP) levels show no differences. (D) Ridgeline plot showing the expression distribution of GSEA results of 736 proteins. (E) Gene Ontology (GO) enrichment of 570 DEGs between the two PYRclusters; "up" means that these pathways of PYRcluster2 were upregulated when compared with PYRcluster1, and "down" means that these pathways were downregulated. ns, no significance.

Supplementary figure 2

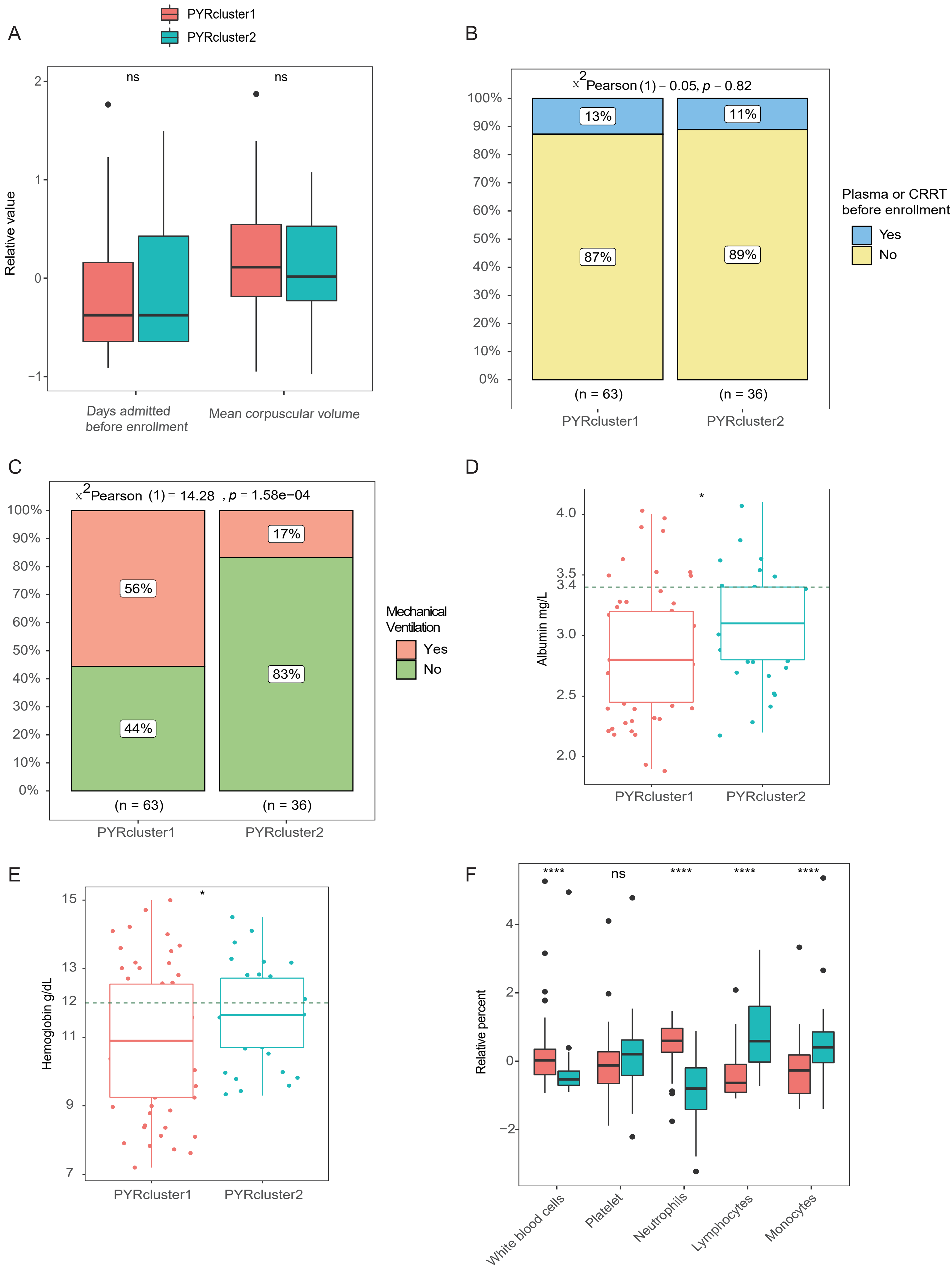

Figure 3: (A) Days admitted before enrollment and mean corpuscular volume of the 2 PYRclusters. (B, C) Percentage of replacement therapy (pre-enrollment) (B) or mechanical ventilation (C) patients in the 2 PYRclusters. (D, E) Blood levels of albumin (D) or hemoglobin in blood. (E) Relative percentage of different cells based on the hemogram results in the 2 PYRclusters. *p < 0.05, **p < 0.01, ***p < 0.001, ****p < 0.0001, ns, no significance (Wilcoxon signed-rank test).

Supplementary figure 3

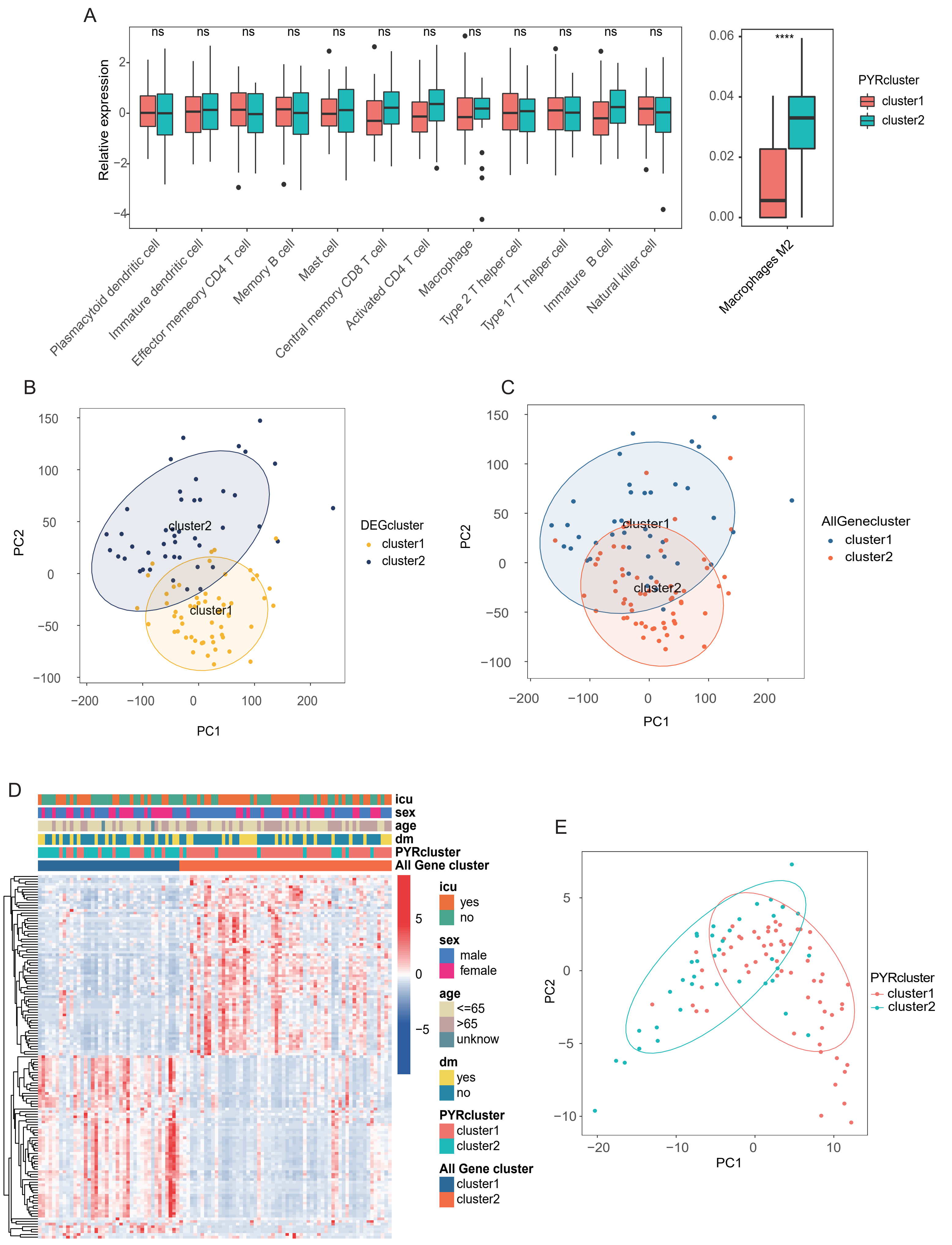

Figure 4: (A) Leukocyte counts, with no significant differences between PYRclusters. (B) PCA of DEGclusters. (C) PCA of All-Gene clusters. (D) DEGs between All-Gene clusters; once these new DEGs between different gene clusters were determined, their heatmap was plotted, showing varied clinical data in the annotation. (E) PCA used to construct the pyrscore.

Supplementary figure 4

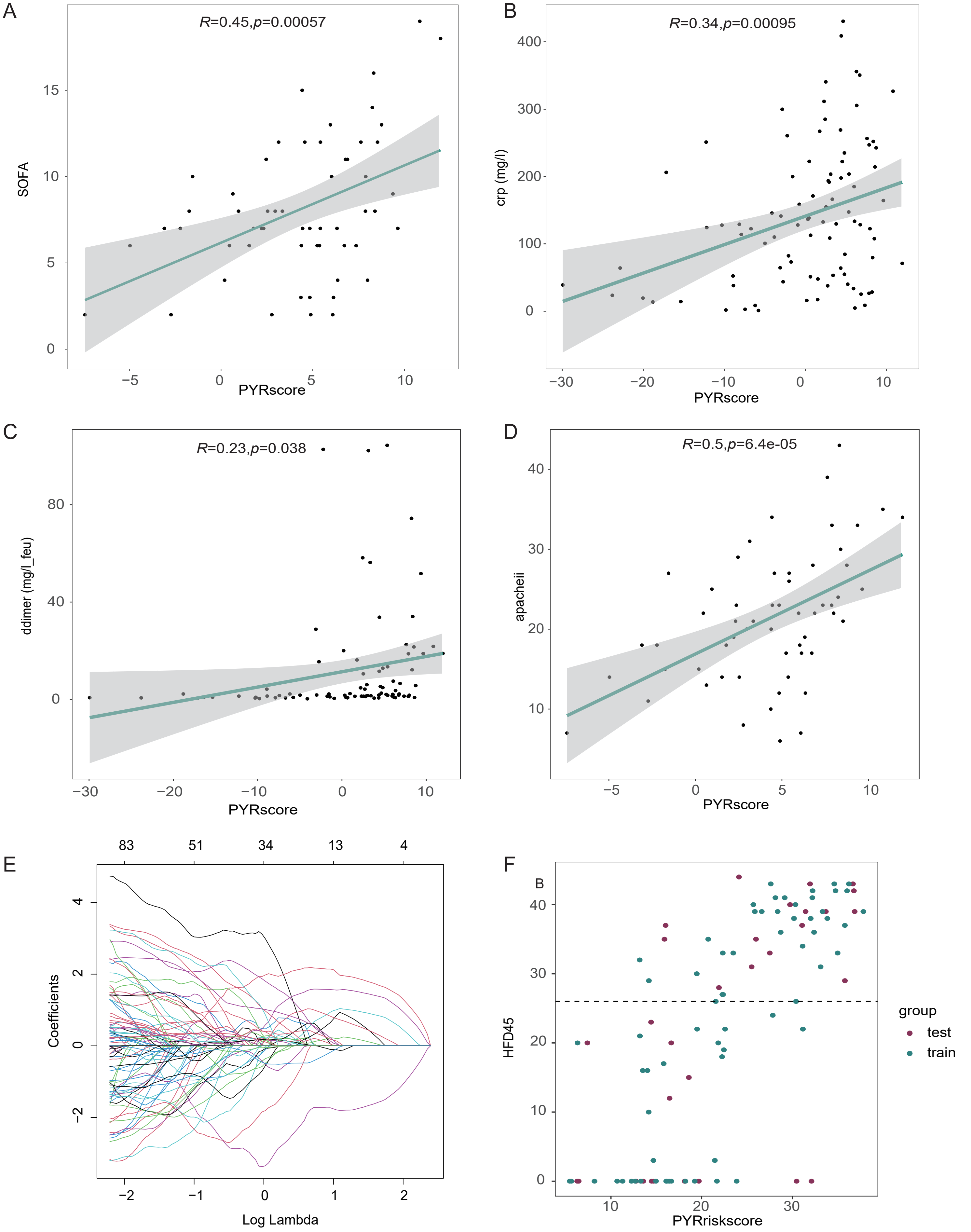

Figure 5: (A, B, C, D) Pearson product-moment correlations between pyrscore and sofa score (A), CRP levels (B), D-dimer levels (C), and APACHE-II (D), with the R value representing the Pearson product-moment correlation coefficient, and the grey area representing the 95% confidence interval for the linear fit. (E) LASSO coefficient profiles. (F) Correlations between PYRsafescore and HFD45 in different groups. The dotted line represents the median value of HFD45 (26).

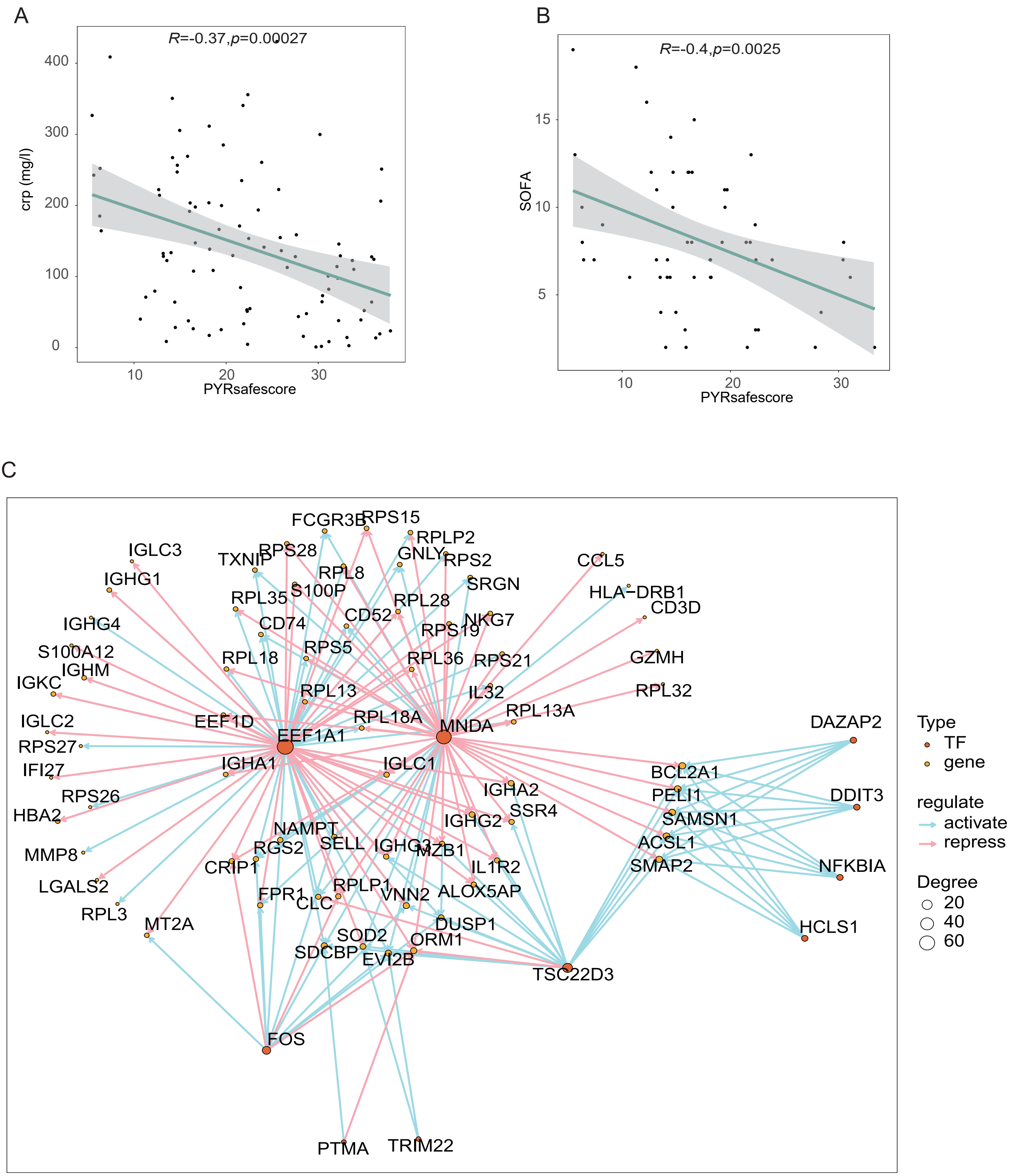

Figure 6: (A, B) Pearson product-moment correlations between PYRsafescore CRP levels (A) and SOFA (B), with the R value representing the Pearson product-moment correlation coefficient, and the grey area representing the 95% confidence interval for the linear fit. (C) Transcription factors regulatory network of PYRcluster2; "degree" means the number of edges connected to each node.

Supplementary figure 6
